# Crosstalk of Intercellular Signaling Pathways in the Generation of Midbrain Dopaminergic Neurons In Vivo and from Stem Cells

**DOI:** 10.3390/jdb7010003

**Published:** 2019-01-15

**Authors:** Claude Brodski, Sandra Blaess, Juha Partanen, Nilima Prakash

**Affiliations:** 1 Department of Physiology and Cell Biology, Zlotowski Center for Neuroscience, Faculty of Health Sciences, Ben-Gurion University of the Negev, Be’er Sheva 84105, Israel; 2 Institute of Reconstructive Neurobiology, University of Bonn Medical Center, 53127 Bonn, Germany; 3 Faculty of Biological and Environmental Sciences, FIN00014-University of Helsinki, P.O. Box 56, Viikinkaari 9, FIN-00014 Helsinki, Finland; 4 Department Hamm 2, Hamm-Lippstadt University of Applied Sciences, 59063 Hamm, Germany

**Keywords:** dopamine, neuron, FGF8, SHH, WNT, BMP, Parkinson’s disease, pluripotent stem cells, iPSC

## Abstract

Dopamine-synthesizing neurons located in the mammalian ventral midbrain are at the center stage of biomedical research due to their involvement in severe human neuropsychiatric and neurodegenerative disorders, most prominently Parkinson’s Disease (PD). The induction of midbrain dopaminergic (mDA) neurons depends on two important signaling centers of the mammalian embryo: the ventral midline or floor plate (FP) of the neural tube, and the isthmic organizer (IsO) at the mid-/hindbrain boundary (MHB). Cells located within and close to the FP secrete sonic hedgehog (SHH), and members of the wingless-type MMTV integration site family (WNT1/5A), as well as bone morphogenetic protein (BMP) family. The IsO cells secrete WNT1 and the fibroblast growth factor 8 (FGF8). Accordingly, the FGF8, SHH, WNT, and BMP signaling pathways play crucial roles during the development of the mDA neurons in the mammalian embryo. Moreover, these morphogens are essential for the generation of stem cell-derived mDA neurons, which are critical for the modeling, drug screening, and cell replacement therapy of PD. This review summarizes our current knowledge about the functions and crosstalk of these signaling pathways in mammalian mDA neuron development in vivo and their applications in stem cell-based paradigms for the efficient derivation of these neurons in vitro.

## 1. Introduction

**Figure 1:**
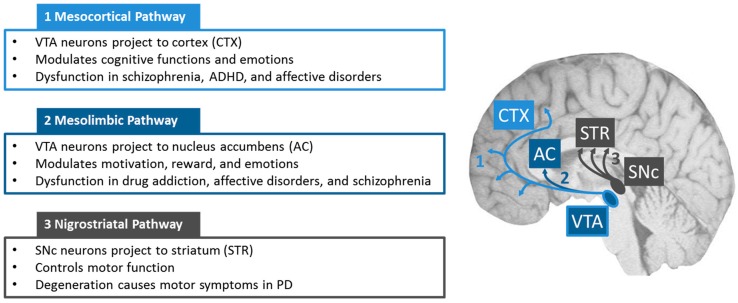
The three dopaminergic pathways originating in the midbrain. Projections, main functions, and major disorders associated with each pathway are listed on the left-hand side. A schematic depiction of the corresponding human dopaminergic pathways is shown on the right-hand side.

The major dopaminergic (DA) neuronal population of the mammalian brain is located in the ventral midbrain (VM) [1]. Confined to a relatively small territory within the VM, mDA neurons are organized into three cell groups, the retrorubral field or A8 group, the substantia nigra pars compacta (SNc) or A9 group, and the ventral tegmental area (VTA) or A10 group [1]. Neurons of the VTA project to the prefrontal cortex to form the mesocortical pathway, which is important for cognition (Figure 1) [2]. Impairment of the DA output to the prefrontal cortex has been implicated in schizophrenia and attention deficit hyperactivity disorder (ADHD) [3,4]. VTA neurons also send their axons to limbic structures to form the mesolimbic pathway linked to motivation, reward, and addiction behaviors. SNc neurons project to the dorsolateral striatum. These SNc projections constitute the mesostriatal pathway, a central modulator of locomotor activity. Reduced striatal DA output due to the degeneration of the SNc DA neurons is the major cause of motor symptoms observed in PD (Figure 1).

The mDA neurons show a remarkable diversity, which has been recognized only recently [5,6,7]. This diversity has been described in terms of their morphological characteristics [8], gene expression pattern [9], electrophysiological features [10], and their connectivity [11]. Such significant differences are determined during the development of the mDA neurons. The molecular mechanisms that underlie mDA progenitor proliferation, specification, and migration, though not yet fully understood, appear to be critical in creating different subsets of these neurons.

The central nervous system in mammals develops from the embryonic neural tube, which is initially divided into four parts: forebrain (prosencephalon), midbrain (mesencephalon), hindbrain (rhombencephalon), and spinal cord. Morphogens secreted from specific organizing centers within or nearby the developing neural tube guide the initial patterning of these regions and provide the neural stem cells (NSCs) with positional information that directs their development according to their location. The mDA neurons develop under the influence of the midbrain FP, containing SHH-, WNT-, and BMP-secreting cells, and the IsO at the MHB, secreting WNT and FGFs from its rostral and caudal border, respectively [12].

Following the induction of the midbrain during embryogenesis [13], a distinct mDA progenitor domain is specified within the midbrain FP. In this domain, radial glia-like neural progenitors divide symmetrically to expand their pool and switch to asymmetric (neurogenic) divisions at the onset of neurogenesis ([14,15] reviewed in References [9,12,16]). The balance between self-renewal and cell cycle exit of the mDA neural progenitors, and the generation of the appropriate numbers of postmitotic progeny, is critical for the proper formation of mDA neurons. So far, four major signaling pathways activated by FGFs, SHH, WNTs, and BMPs have been identified in mammals to control the proliferation and specification of mDA progenitors in vivo (reviewed in References [9,12,17,18,19]). Downstream of these signaling pathways, expression of a series of transcription factors (TFs) is activated to regulate progenitor cell responsiveness to these morphogens (OTX2, LMX1A/B, and FOXA1/2; [20,21,22,23,24,25,26,27,28]), neurogenesis (MSX1/2 and NGN2; [23,29,30]), and mDA neuron differentiation and survival (EN1/2, NURR1, PITX3; [31,32,33,34,35,36,37,38,39]).

Parkinson’s Disease (PD) is a prevalent and highly debilitating neurodegenerative disease [40]. For still not fully understood reasons, the SNc mDA neurons are particularly vulnerable to degeneration and their loss is a neuropathological hallmark of PD [12]. Currently, there is no cure for PD and treatments using the DA precursor L-DOPA or DA receptor agonists can only ameliorate the symptoms, but do not stop the progression of the disease. One of the most promising new approaches to treat PD is cell replacement therapy using human pluripotent stem cell (PSC)-derived mDA neurons, for which the first clinical trials are currently underway [41]. Moreover, stem cell-derived mDA neurons are also becoming an indispensable tool for PD modeling and drug screening. However, the differentiation of stem cells to mDA neurons requires the knowledge of the exact molecular mechanisms directing the embryonic development of mDA neurons in vivo. Current in vitro differentiation protocols for mDA neurons are based on the activation of the three signaling pathways, FGF, SHH, and WNT, which regulate the formation of mammalian mDA neurons in vivo [12,42,43,44]. Therefore, understanding how these pathways interact is expected to critically increase the yield and quality of stem cell-derived mDA neurons.

In this review, we will focus on the generation of mammalian mDA neurons. To investigate the development of these neurons in vivo, mouse mutants are the prime model. Conditional mouse mutagenesis provides extensive possibilities to determine specific effects of the inactivation of genes of interest with high spatiotemporal precision. However, caveats and drawbacks of these models need to be taken into consideration. The driver mouse lines do not always lead to a complete inactivation of the corresponding gene and may result in mosaicisms [45]. Insights from mouse mutants cannot necessarily be applied directly to the human situation, and direct pathway interactions are not easily determined [46]. Moreover, mouse models of PD generally do not recapitulate all features of the human disease [47], which is one main reason why patient-specific, stem cell-derived mDA neurons have gained substantial interest in recent years. To study the generation of these neurons in vitro, different mouse as well as human stem cells are widely used and will therefore be discussed in the following sections in more detail.

## 2. Signaling Pathways in Midbrain Dopaminergic Neuron Generation In Vivo and In Vitro

### 2.1. FGFs/FGF8 Signaling

#### 2.1.1. FGF Signaling Pathway in the Embryonic Midbrain

**Figure 2:**
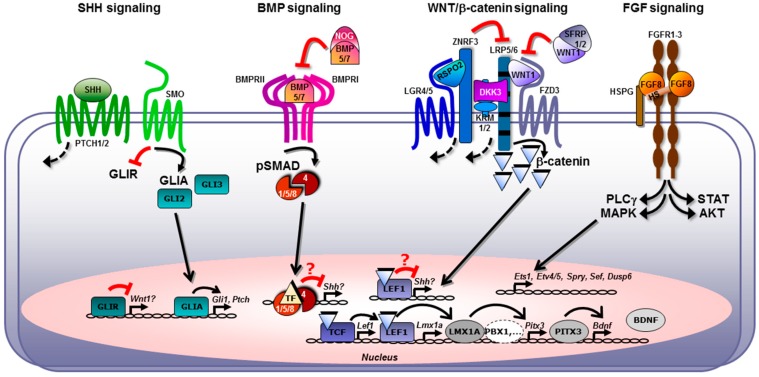
Signaling pathways in mDA neuron development. Schematic and simplified depiction of the main signaling pathways implicated in the generation and maintenance of mDA neurons in the mammalian embryo, SHH, BMP, WNT/β-catenin, and FGF (from left to right), and their proven or yet unknown gene targets in the cell nucleus. Arrows indicate activation, crossbars indicate inhibition of the corresponding pathway or target molecule/gene. Stippled curved lines indicate endocytosis of the corresponding ligand-receptor/co-receptor complex. Question marks denote a still unclear target gene or mechanism in the corresponding pathway during mDA neuron development. See text for details and abbreviations.

Fibroblast growth factors (FGF1-22) are a family of secreted signaling molecules [48]. In a tissue, spreading and signaling of most FGFs are modulated by their interaction with the extracellular matrix, in particular heparan sulfate proteoglycans. FGFs affect their target cells by binding to cell surface receptors belonging to the receptor tyrosine kinase family (FGFR1–FGFR4). This results in activation of multiple downstream signal transduction cascades, in particular the mitogen-activated protein kinase (MAPK), phosphoinositide-3-kinase/protein kinase B (PI3K/AKT), phospholipase C gamma (PLCγ), and signal transducers and activators of transcription (STAT) pathways [48] (Figure 2). In addition to these canonical signal transduction mechanisms, signaling via nuclear FGFR localization has been suggested [49]. The initial transcriptional response to the FGF stimulation is similar in many cell types and often includes activation of expression of members of the Ets family TFs, such as ETV4 (PEA3) and ETV5 (ERM), as well as feedback signaling modulators, including Sprouty (SPRY) and dual specificity phosphatase (DUSP) gene products (Figure 2). The cellular response to the activation of the FGF-stimulated intracellular signaling cascade and the later FGF-induced gene expression changes are highly dependent on the type of the target cell. FGFs have been shown to regulate cell survival, proliferation, differentiation, migration, metabolism, axon guidance, and subcellular differentiation, such as synaptogenesis.

Several FGF family members regulate the development of the embryonic midbrain, including its FP, which gives rise to mDA neurons. In particular, FGF8, expressed early in the entire rhombomere 1 and later in the posterior (*Gbx2*-positive) part of the IsO, is highly important for midbrain development [50]. At the IsO, FGF8 is produced as two alternatively spliced isoforms, FGF8a and FGF8b. Of these, FGF8b has a higher receptor binding affinity and appears to carry out the majority of the signaling function [51]. Related FGFs, FGF17 and FGF18, are also expressed in the IsO in an FGF8-dependent fashion, but, in contrast to FGF8, in both the anterior (*Otx2*-positive) and posterior (*Gbx2*-positive) parts of the IsO. They appear to support the FGF8 functions but have weaker signaling capacity compared to FGF8b [51,52]. Other FGF family members have also been detected in the developing midbrain in different patterns. For example, FGF15 is expressed in the laterodorsal region, but not close to the MHB [53,54]. However, studies on FGF protein localization, detection of FGF receptor binding activity and expression of the proximal FGF target genes all suggest that, in the early embryonic midbrain, FGF growth factors form both molecular and activity gradients increasing towards the IsO [55,56].

Of the signal transducing FGF receptors, FGFR1, FGFR2, and FGFR3 are expressed in the embryonic midbrain. FGFR1 is broadly expressed throughout the midbrain neural progenitors, whereas FGFR2 and FGFR3 are not detected close to the MHB. Consistent with their patterns of expression, FGFR1, FGFR2, and FGFR3 redundantly receive FGF signals, FGFR1 being the main receptor close to the IsO [57,58]. FGFR function is regulated, both positively and negatively, by several proteins. Interestingly, the expression of many of the genes encoding for such regulatory proteins is stimulated by the FGF signaling pathway in the embryonic midbrain [59]. The positive regulators include canopy FGF signaling regulator 1 (CNPY1), which promotes FGFR maturation in the endoplasmic reticulum, and fibronectin leucine rich transmembrane protein 3 (FLRT3), a transmembrane protein shown to form a complex with FGFR [60,61,62,63]. On the other hand, FGF-induced negative feedback signal regulators, such as interleukin 17 receptor D (SEF/IL17RD), Sprouty related EVH1 domain containing (SPRED), SPRY and DUSP proteins, regulate various steps in the intracellular signal transduction pathway (reviewed in Reference [64]). The reason why FGF signaling induces both positive and negative feedback regulation remains largely unclear. It may regulate the timing of active FGF signaling, suggested to be important for the signaling outcome [65]. The negative feedback regulators also provide another means to shape the FGF signaling gradient in the developing tissue.

#### 2.1.2. FGF-Regulated Developmental Processes in the mDA Neuron Progenitors

Already 20 years ago, the discovery of FGF8 as an important signal of the IsO led to the demonstration of its requirement and potency in instructing the development of mDA neurons [50,66]. In the embryonic midbrain, FGF signaling has been suggested to control several developmental processes discussed below. Although many of these studies focused on the more dorsal regions of the midbrain, it is likely that these processes are also FGF-regulated in the developing mDA neuron progenitors in the VM.

After neural induction, FGF signaling is instrumental for both establishment and maintenance of the IsO at the MHB [50]. FGF8 is an integral part of a signaling network at the IsO, where the expression of FGF8 and WNT1 are dependent on each other (see Section 2.5.1). This fact also makes understanding of the direct versus indirect effects of FGF signaling more challenging in the in vivo models.

Following the establishment of the IsO, FGF8 signaling is important for the maintenance of the neural progenitor viability. Inactivation of FGF8 or FGFRs results in an increase of apoptotic cell death in the early embryonic midbrain neuroepithelium [57,67]. Although more prominent in the dorsal (alar) midbrain, increased apoptosis was also detected in the VM of the *Fgfr* mutants, where it occurred prior to the onset of mDA neuron differentiation [57].

FGF signaling regulates anterior–posterior (A/P) patterning and compartmentalization of the midbrain [68,69]. Strong FGF8b signaling can transform the midbrain tissue into rhombomere 1/isthmus identity, positive for *Gbx2* expression [70,71,72]. This may correspond to the observations that in rat explant cultures, FGF4 stimulation, likely resulting in a robust FGFR activation, yields serotonergic neurons characteristic for the ventral hindbrain [66]. Lower levels of FGF signaling from the IsO appear important for the A/P patterning of both the dorsal midbrain and the VM [73,74,75]. During mDA neuron development, early postmitotic neuronal precursors expressing tyrosine hydroxylase (TH) are produced in a relatively broad A/P region, starting from the diencephalic p3 domain and extending posteriorly up to the MHB. Recent fate mapping and transcriptional profiling studies suggest that the mDA neurons arise from progenitors derived from *En1* expressing cells, which, in addition to the midbrain, encompass the basal region of the diencephalic p1 and p2 domains (this is in contrast to the alar region, where the *Pax6*/*En1* boundary defines the diencephalon (p1)/midbrain border) [76,77]. In turn, the basal p3 domain belongs to the *Dbx1* cell lineage and gives rise to neurons in the subthalamic and premammillary nuclei, which are non-dopaminergic, yet share the expression of many genes active in mDA precursors [76,78]. Although derived from the *En1* expressing cell lineage, the basal p1 and p2 progenitors appear to later mostly downregulate *En1* and *En2* expression [75]. The TH-expressing precursors derived from these regions are also negative for the expression of *En1*, *En2*, and many other mDA markers detected in the embryonic midbrain. This difference appears to be due to FGF signaling in the midbrain. If FGFRs are inactivated in the midbrain neuroepithelium, both the ventral progenitors and the TH-expressing precursors derived from them transform to a ventral diencephalic identity, including the loss of *En1* and *En2* expression [75]. In the *Fgfr* mutant embryos, TH expression appears to be later downregulated without apparent cell death. Similarly, in conditional *Fgf8* mutant mice, TH-expressing precursors are initially produced in the embryonic midbrain, but TH-positive mDA neurons are not detected in the perinatal brain [67,79]. Whether the loss of TH expression reflects the normal fate of the diencephalic p1/p2-derived TH-positive precursors remains unclear. Understanding the contribution of the diencephalic TH-expressing precursors to the mDA nuclei would require fate-mapping tools able to distinguish the basal midbrain and p1/p2 domains. The early embryonic brain patterning generates two main types of mDA neurons along the A/P axis of the midbrain and diencephalon, postnatal development extending this diversity to at least five molecularly distinct subtypes [46,80]. However, both of the embryonic mDA neuron subgroups appear to be molecularly related to the midbrain-derived precursors.

In addition to the regional identity, both gain-of-function (GOF) and loss-of-function (LOF) studies suggest that FGF signaling regulates the balance between neural progenitor maintenance and neurogenic cell cycle exit in the embryonic midbrain, including the developing mDA neurons [56,81]. In the neural progenitors, the basal process may transduce the basal lamina-derived FGF signals to promote *Hes1* and *Sox3* expression, which in turn inhibit proneural gene expression and neurogenic cell cycle exit [56,82]. When FGF signaling is inactivated, *Hes1* and *Sox3* expression is downregulated and the embryonic VM precociously generates TH-positive precursors. Consistently, the early production of TH-expressing precursors is also increased in *Hes1* mutant embryos [83]. The exact molecular identity of the FGF signal promoting neural progenitor maintenance remains unclear. Nevertheless, it has been shown that, compared to neuroepithelial patterning, lower signaling levels stimulated by FGF8a, FGF17, or FGF18 can promote progenitor proliferation [72,84]. Interestingly, some of the FGFs appear to have antagonistic functions. In particular, FGF15, expressed throughout the dorsolateral midbrain, promotes neurogenic differentiation rather than progenitor proliferation [54]. The mechanism behind the apparently opposite functions of FGF8 and FGF15 in progenitor regulation remains unclear.

During later development of the mDA system, FGFs have additional functions, including axon guidance [85]. Interestingly, the mature mDA neurons express certain FGF family members, such as FGF20, possibly regulating their survival and other cellular functions [86,87,88,89]. Notably, the human *FGF20* gene locus has been associated with PD [90], although the mechanisms behind this remain unclear.

#### 2.1.3. FGF Signaling Promotes mDA Neuron Differentiation In Vitro

In vitro, FGF signaling regulates the proliferation and differentiation of NSCs, including embryonic neural progenitor cells isolated from the midbrain [91,92]. Moreover, FGF signaling is required for mDA neuron development and exogenous FGF8 induces mDA neuron differentiation in neural explants [66]. These findings, together with the knowledge of FGF functions during midbrain development in vivo, motivated the design of protocols for mDA neuron differentiation from neural progenitors, embryonic stem cells (ESCs), induced PSCs (iPSCs), or reprogrammed somatic cells, such as fibroblasts [12,93,94,95,96]. In the cell culture, exogenous FGF stimulation may mimic signaling events at various developmental steps, including PSC neuralization, and NSC patterning, proliferation and maintenance. Underlining the importance of fine-tuning the neural patterning, a recent study shows a correlation between the expression of posterior midbrain genes, including many FGF target genes, and the success of intracerebral grafting of ESC-differentiated mDA precursors [97]. Furthermore, patterning of ESC-derived VM progenitors with a timed delivery of FGF8 resulted in posterior midbrain-type mDA neuron precursors, which in turn gave rise to dopamine-rich grafts able to alleviate symptoms in a rat model of PD [97,98,99]. These results are consistent with the observed requirement of FGF signaling in patterning of the VM in vivo. Combined with the modulation of the other signaling pathways, in particular SHH and WNT, FGF signaling may be used for more precise patterning of the neural progenitor cells to give rise to cultures of correctly specified mDA neurons and their subtypes, with little contamination of related cell lineages [76,97].

### 2.2. SHH Signaling

#### 2.2.1. The SHH Signaling Pathway

Hedgehogs are secreted morphogens with multiple functions during embryonic development. In vertebrates, there are three family members: Desert Hedgehog (DHH), Indian Hedgehog (IHH), and Sonic Hedgehog (SHH) [100,101]. SHH is the Hedgehog protein that is essential for the developing nervous system [102]. The core pathway through which SHH (as well as IHH and DHH) signaling is transduced in mammals consists of the twelve-pass transmembrane receptor Patched (PTCH), the G-protein coupled receptor Smoothened (SMO) and three Gli TFs (glioma-associated oncogene; GLI1-3) [100] (Figure 2). In the absence of SHH, the activity of SMO is suppressed by PTCH [103], and GLI2 and GLI3 are proteolytically cleaved to repressor forms (GLIR) [104,105]. GLI3R is the main repressor downstream of SHH signaling and inhibits the transcription of SHH target genes [106,107]. In the presence of SHH, the inhibition of SMO by PTCH is released [103]. As a consequence, the formation of GLIR proteins is attenuated and GLI2 and GLI3 are present as full-length activators (GLIA) [104,105] (Figure 2). GLI2A has the main activator role downstream of SHH, GLI3A has only a minor function as activator [108,109,110]. GLIA in turn induce the transcription of GLI1, a constitutive activator in the SHH pathway that enhances the activating side of the SHH signaling pathway [108,109,111]. Another target gene of activated SHH signaling is PTCH, adding a negative feedback loop to the signaling pathway [112]. Both GLI1 and PTCH expression have been used as readout for activated SHH signaling [113,114] (Figure 2). An important aspect of SHH signaling is that the key components of the SHH signaling pathway, PTCH, SMO and the GLI proteins are localized in and around the primary cilium. Primary cilia are a single copy, non-motile membrane protrusion present in most mammalian cells and are thought to serve as a receiver of extracellular signals. The regulated transport of PTCH, SMO, and GLI proteins in and out of the primary cilia is critical for the processing of GLI proteins into their activator and repressor forms [115]. Thus, the loss of functional primary cilia results in phenotypes similar to the ones observed when both GLIA and GLIR function are inactivated (i.e., in *Gli2*/*3* double mutants) [115].

In addition to this core signaling pathway, there are several PTCH co-receptors that are required for the binding of SHH with high affinities, such as growth arrest-specific 1 (GAS1), CAM-related/downregulated by oncogenes (CDO), and brother of CDO (BOC) [116,117]. Additional components downstream of PTCH and SMO include SUFU (suppressor of fused), which regulates SHH pathway activity negatively; KIF7, which is important for the transport of GLI2/3 through the primary cilium as well as several protein kinases (protein kinase A (PKA), casein kinase 1 (CK1), and glycogen synthase kinase 3β (GSK3β)), that phosphorylate GLI2/3 and thus mark them for proteolytical processing [100].

#### 2.2.2. Expression of Shh Pathway Components in the Ventral Midbrain

Midbrain dopaminergic neurons are derived from a LMX1A/B (LIM homeobox transcription factor 1)- and FOXA1/2 (forkhead/winged helix transcription factors)-positive progenitor domain that is established around day 9.5 of embryonic development (E9.5) at the ventral midline of the mesencephalon. SHH and its downstream pathway components show a dynamic expression in the mesencephalic ventral midline. In the developing murine VM, *Shh* is initially expressed in the notochord that underlies the forming neural tube (starting at E7.5). Notochord-secreted SHH induces GLI2A in the midline of the neural tube (the FP) between E8.0 and E8.5. The activation of the SHH signaling pathway results in the expression of *Gli1* and *Foxa1*/*2* in the FP. FOXA1/2 are in turn necessary to induce expression of *Shh* in FP cells around E8.5 [118,119,120,121,122]. *Shh* expression expands over the following days encompassing not only the FP but also part of the adjacent basal plate (BP; up to E10.5) and thus the entire LMX1A-positive mDA progenitor domain. At E11.5, *Shh* expression is downregulated at the ventral midline, leaving two stripes of cells lateral to the ventral midline that continue to express *Shh* in the subsequent days and that partially overlap with the mDA progenitor domain [120,122,123,124,125]. After *Shh* expression is induced in the ventral midline, *Gli2* is downregulated in the SHH-positive FP and BP areas, but expression is maintained in the lateral and dorsal progenitors in the midbrain. *Gli3* expression is restricted to the alar plate after E8.0. Both *Gli1* and *Ptch* are strongly expressed in a narrow domain just lateral to the SHH-expressing domain but are absent from the SHH-expressing domain itself. Thus, with the expansion of the *Shh*-expressing domain between E8.0 and E12.5, *Gli1* and *Ptch* expression domains move further lateral within the BP and only overlap with the mDA progenitor domain before E10. *Smo* is expressed throughout the VM [121,122,123,124,125,126,127]. As for the PTCH co-receptors, CDO expression in the ventral midline has been shown at E9.5 and E12.5 [128,129], while GAS1 expression is restricted to the dorsal midbrain and is only detected at E12.5 and not at later stages [130]. BOC expression has not been analyzed in the VM.

In summary, all the components necessary for activating the canonical SHH pathway (SMO, PTCH, GLI1, GLI2) are expressed in the mDA progenitor domain during early neural development but are downregulated after E9.5 (except for SMO). Moreover, neither GLI1 nor PTCH are detected in differentiated mDA neurons, indicating that canonical SHH signaling does not play a role in differentiated mDA neurons [127,130]. SMO is maintained in differentiated mDA neurons where it functions in non-canonical signaling during axon pathfinding [131].

#### 2.2.3. Fate Mapping of SHH-Expressing and SHH-Responding Progenitors in the VM

The dynamic expression patterns of *Shh* and *Gli1* in the mDA progenitor domain have been used to determine whether different mDA progenitor domains give rise to specific neuronal subpopulations in the VM. A genetic fate-mapping approach that labels all *Shh*-expressing cells in a permanent manner shows that most (if not all) mDA neurons are derived from *Shh*-expressing cells. In addition, *Shh*-expressing cells also generate non-mDA neurons in a number of other VM nuclei, including neurons in the red nucleus [124,132,133]. Besides providing insight into the ventral midline origin of mDA neurons, these results in combination with additional fate-mapping studies demonstrated that the mesencephalic FP is neurogenic, in contrast to FP cells in the spinal cord and hindbrain, which act as organizers for the surrounding tissue but do not give rise to neurons [14,132,134,135]. To generate fate-maps with temporal and spatial resolution that reflect the dynamic expression of *Shh* and the SHH signaling readout *Gli1*, genetic inducible fate-mapping has been used to follow the fate of *Shh*-expressing and *Gli1*-expressing (equivalent to SHH-responding) mDA progenitors and their descendants at various time points of development. These fate-maps show that cells within the mDA progenitor domain (that express *Gli1* at E7.5 or *Shh* at E8.5) preferentially give rise to SNc neurons, whereas cells that express *Gli1* or *Shh* later in development (at E9.5 or E11.5, respectively) almost exclusively give rise to VTA neurons [123,136]. After E9.5, *Gli1*-expressing cells do no longer give rise to mDA neurons, since the *Gli1* expression domain lies outside of the mDA progenitor domain. The contribution of *Shh*-expressing cells to mDA neurons tapers off after E11.5, probably because the neurogenic potential of the progenitors decreases [123,136,137]. The existence of two mDA progenitor domains with distinct fate potential was corroborated by the analysis of TF expression domains in the mDA progenitor domain and in differentiated mDA neurons: OTX2 is expressed at high levels in the lateral mDA progenitor domain and is largely restricted to the VTA in differentiated mDA neurons. The medial mDA progenitor domain expresses SOX6, this TF is expressed in differentiated mDA neurons of the SN [138].

#### 2.2.4. In Vivo Function of the SHH Signaling Pathway

The first evidence that SHH signaling plays an important role in the induction of mDA neurons came from in vitro studies and ectopic expression of *Shh* or *Gli1* in the midbrain of transgenic mice [66,139,140]. This was corroborated with LOF experiments in the mouse: inactivation of the SHH signaling pathway in *Shh* null or *Gli2* null mutants results in the loss of most VM structures, including the (almost) complete absence of the mDA progenitor domain and the mDA neuronal population [110,121,141].

Conditional gene inactivation (cKO) of *Smo*, *Gli2*, *Gli2*/*Gli3*, or *Shh* around E8.5 in the midbrain and anterior hindbrain using *Engrailed1* (*En1*)-*Cre* mice does not result in the loss of the entire mDA progenitor domain [120,125,142,143], likely because the partial induction of the mDA progenitor fate by SHH signaling occurs earlier than E8.5: as described above, cells that respond to SHH (express *Gli1*) are already observed in the ventral midline around E7.5 [123,136,144]. Notably, the phenotypes vary in their severity depending on which component of the pathway has been conditionally inactivated. In *Smo* cKO mutants, only very few mDA progenitors are induced and the number of mDA neurons is severely reduced [120]. In *Shh*, *Gli2*, or *Gli2*/*Gli3* cKO mutants, the numbers of mDA progenitors and mDA neurons are reduced by half compared to the wild-type [120,125,142,143]. The difference between *Smo* and *Gli2* cKO or *Gli2*/*Gli3* cKO mutants is likely due to an increase in GLIR in *Smo* cKO mutants, which results in massive apoptosis in the basal and alar plate [120]. In the *Shh* cKO mice, the phenotype is milder than in the *Smo* cKO mice, since SHH signaling appears to be still weakly activated judging by the faint *Gli1* and *Ptch* expression in the BP [125]. The partial activation of the pathway suggests that SHH is still present in the extracellular space of the VM. One potential source for SHH may be the BP of the adjacent posterior hypothalamus [145].

Mutant (cKO) mice, in which *Smo* was inactivated using *Shh-Cre*, had mild and transient phenotypes in mDA progenitors and during mDA neurogenesis that do not affect the overall number of mDA neurons [122,124]. GOF experiments confirm the minor role of SHH signaling in mDA development at this time point: expression of a constitutively-active SMO in *Shh*-expressing cells results in only a transient increase in the size of the mDA progenitor domain [124]. Finally, inactivation of *Smo* or *Gli2* after E10.5 using *Nestin-Cre* does not result in any discernable phenotype in the mDA progenitor domain or in the number of mDA neurons [120]. However, inactivation of *Smo* at this time point leads to axonal pathfinding defects in a small subset of mDA neurons [131].

These gene inactivation approaches have also been used to explore whether SHH signaling might contribute to setting up distinct mDA progenitor domains. In *Gli2* cKO mice, the 50% reduction in the size of the mDA progenitor domain is mainly due to the loss of lateral mDA progenitors. Consistent with the genetic inducible fate-mapping data that show that these progenitors contribute preferentially to VTA neurons (see Section 2.2.3.), the reduction in mDA neurons in the adult brain of these mouse mutants can be largely attributed to a loss of VTA DA neurons [143]. Inactivation of the PTCH co-receptor *Cdo* also results in a specific effect on VTA DA neurons. In *Cdo* null mice, proliferation in the VM ventricular zone is increased at E12.5 compared to controls, and in postnatal brains an increased number of VTA (but not SNc) DA neurons has been reported [129]. However, another study reports a severe decrease in the number of mDA neurons in *Cdo* null mice at E13.5 [128]. This may suggest that there is a transient delay in neurogenesis in *Cdo* null mice that is later (over)compensated. A detailed birthdating analysis in mutants in which *Smo* was inactivated with *Shh-Cre* shows transient effects of SHH signaling inactivation on the generation of VTA neurons: a transient increase in mDA progenitor proliferation at E11.5 is correlated with an increased contribution to the VTA, while at E13.5, increased cell cycle exit results in a depletion of proliferating mDA progenitors and reduced contribution to the VTA. The combination of transient increase and later depletion leads to no obvious change in the overall number of mDA neurons [122].

The importance of primary cilia for SHH signaling has also been demonstrated in the context of mDA progenitor induction. In a mouse mutant carrying two hypomorphic alleles for a gene encoding one of the ciliary transport proteins, IFT88, primary cilia function is defective but SHH signaling is not completely abolished. Small, rosette-like clusters maintain their ability to express *Shh* and/or *Gli1*, and a few mDA neurons are induced [142]. Conditional inactivation of *Ift88* using *En1-Cre* mice results in a severe loss of primary cilia and in inactivation of the SHH signaling pathway before E9.5, and a phenotype in the mDA progenitor domain and in differentiated mDA neurons that is comparable to *Gli2* cKO or *Gli2*/*Gli3* double-cKO mutants [142]. Conditional inactivation of *Kif3a*, encoding a kinesis motor protein important for cilium assembly and maintenance, abolishes primary cilia and SHH signaling in the VM only after E9.5 and results in a transient phenotype, in which the mDA progenitor domain is reduced but the number of differentiated mDA neurons in the prenatal brain is comparable to controls [146].

The combined insights from gene expression studies, fate mapping and gene inactivation approaches demonstrate that there is a critical period in which SHH plays a role in the development of mDA progenitors that ends around E9.5 in the mouse.

#### 2.2.5. Function of SHH Signaling in the Generation of mDA Neurons from PSCs

Generation of mDA neurons from PSCs for cell replacement strategies in PD and/or disease modelling has been pursued for many years. Initially, these efforts were hampered, since the obtained neurons did not display all the principal characteristics of authentic mDA neurons (e.g., they lacked FOXA2 expression) and showed poor survival after transplantation [147]. The finding that mDA neurons are generated from FP-like, SHH-expressing cells was crucial for setting up protocols that allow efficient and high-yield production of mDA neurons from human PSCs. Since FP cells are established as a cell lineage distinct from BP neuronal progenitors during early neural development [148], this was an essential step in generating authentic mDA neurons in vitro. The Studer lab first implemented this concept to generate FP-type cells from PSCs [149]. Fasano and colleagues directly differentiated human ESCs into FP cells by adding high doses of SHH at early stages of a neural differentiation protocol that uses two BMP/transforming growth factor beta (TGFβ) (“dual SMAD”) inhibitors (day 1–9). FP cells were identified based on expression of FOXA2 and secretion of Netrin and SHH. These FP cells had anterior characteristics, but adding retinoic acid, FGF8, or WNT1 together with SHH shifted their fate posteriorly. In particular, addition of WNT1 resulted in FP cells with midbrain phenotypes as assessed by expression of EN1, LMX1B and Corin [149]. These insights were then utilized to generate neurons with the cardinal features of mDA neurons from human PSCs. To this end, the above described FP induction protocol was modified: SHH and FGF8 signaling is activated from day 1–7 of neural differentiation, followed by the activation of WNT signaling from day 3 onwards. After 11 days, this protocol yields midbrain FP cells and after 25 days, neurons with the characteristic expression profile of mDA neurons [43]. A slightly different protocol, in which SHH and WNT signaling were activated from day 0 to day 9 of neural differentiation was established by Kirkeby et al. and also results in the successful generation of authentic mDA neurons [150]. Importantly, in the context of cell replacement strategies, when the generated mDA neurons were transplanted into the brain of rats or monkeys they survived over several months, showed functional integration and improved behavioral outcomes in animals with lesions in the SNc [43,150,151]. Based on these results, protocols have been further refined to generate high yields of mDA neurons from PSCs and the first clinical application of hPSC-derived mDA neurons is now pursued for cell replacement in PD patients [41,99].

### 2.3. WNT Signaling

#### 2.3.1. WNT Signaling Pathways and Mechanisms

After the discovery of an involvement of the FGF8 and SHH signaling pathways in the development of mDA neurons in mammals [66,139] (Section 2.1 and Section 2.2), the prominent role of the WNT signaling pathways in mDA neuron generation in vitro (in the culture dish) and in vivo (in mice) was revealed only a decade later [152,153,154]. Meanwhile, both FGF8/SHH and WNT proteins or agonists are routinely used for the directed differentiation of human PSCs into mDA neurons (reviewed by [155,156]). The distinct WNT signaling pathways known so far in mammals are briefly summarized in the next two paragraphs, but the reader is referred to several recent reviews on the subject for more detailed information [157,158,159,160,161].

The 19 mammalian WNTs are secreted, which are lipid-modified (palmitoylated) glycoproteins that bind to a particular class of seven-pass transmembrane domain proteins, the frizzled (FZD) receptors (10 in mammals) [157] (Figure 2). Mounting evidence has shown that, because of their lipid modification, WNTs are very hydrophobic and short-range signaling molecules rather than classical morphogens. The highly regulated release of WNT molecules from the cell surface or within extracellular vesicles (exosomes), however, also enable the action of WNTs over longer distances [157]. Upon binding to their FZD receptors, WNT signals can be transduced via distinct pathways in vertebrate cells: the best studied pathway is the WNT/beta-catenin (β-catenin, *Ctnnb1*) or “canonical” pathway [159], whereas less is known about the “non-canonical” WNT/planar cell polarity (PCP) and calcium (Ca^2+^) pathways [161]. The hallmark of the WNT/β-catenin pathway is the cytosolic accumulation of “free” (not bound to cadherins) and N-terminally unphosphorylated β-catenin after binding of WNT to its FZD receptor and another single-pass transmembrane protein, the low-density lipoprotein receptor-related protein (LRP) 5/6 co-receptor (Figure 2). This is due to the not yet fully understood inactivation of a so-called “destruction complex” consisting of the scaffolding proteins axin and adenomatosis polyposis coli, the serine-threonine kinases CK1α/δ (Csnk1a/d) and GSK3α/β, and the beta-transducin repeat-containing E3 ubiquitin-protein ligase (βTrCP, Btrc) [157,159]. In the absence of a WNT signal, constitutive and sequential phosphorylation of serine residues in the β-catenin N-terminus by CK1 and GSK3 is recognized by βTrCP, which poly-ubiquitinates β-catenin thereby targeting it for proteasomal degradation. In the presence of a WNT signal and upon inactivation of the destruction complex, stabilized (unphosphorylated) β-catenin in the cytosol is relocated to the cell nucleus, where it binds to members of the high mobility group TF family, including lymphoid enhancer binding factor 1 (LEF1) and the T cell factors TCF7, TCF7L1, and TCF7L2 (Figure 2). In the absence of a WNT signal/nuclear β-catenin, LEF1/TCFs are associated with transcriptional repressors such as groucho/transducin-like enhancer of split proteins, which inhibit the activation of LEF1/TCF-bound WNT/β-catenin target genes. Displacement of these transcriptional repressors by β-catenin and recruitment of co-activators, such as pygopus and B cell CLL/lymphoma 9, to the DNA-bound LEF1/TCF complex leads to the activation of a broad but context-specific variety of WNT/β-catenin target genes. Several of these target genes are involved in the regulation of the cell cycle and proliferation of tissue-specific stem cells, whereas other targets of this signaling pathway participate in the acquisition of a particular cell fate by their progeny. It has therefore been suggested to rename the WNT/β-catenin signaling pathway as the “cell fate” WNT pathway [158]. This signaling pathway is inhibited or at least attenuated by several antagonists, including secreted proteins that either bind and sequester the WNT ligand directly, such as secreted frizzled-related proteins (SFRPs), inactivate the WNT ligand enzymatically, or interact with the LRP co-receptors and other single-pass kringle containing transmembrane proteins (KREMEN1/2) to induce their rapid internalization, such as the dickkopf (DKK1–4) family, thereby inhibiting the formation of an active WNT-FZD-LRP complex [157] (Figure 2). The availability of extracellular WNT proteins and cell surface FZD receptors is also controlled by other transmembrane proteins, such as the WNT N-terminal cleaving metalloproteases TIKI1/2 (*Trabd*) or the ring finger E3 ubiquitin ligases ZNRF3 and RNF43, which ubiquitinate the FZD receptors to promote their internalization and subsequent proteasomal degradation [157]. Notably, the secreted R-spondins (RSPO1–4) act as WNT agonists in this context by binding to the seven-pass transmembrane domain leucine-rich repeat-containing G protein-coupled receptors (LGR4-6) and inhibiting the ZNRF3 and RNF43 E3 ubiquitin ligases, thus augmenting the availability of FZD receptors at the cell surface (Figure 2). The disheveled (DVL) protein is another important component of WNT signal transduction in both the “canonical” and “non-canonical” pathways. Upon WNT binding to the FZD receptor, DVL is hyperphosphorylated and associates with the intracellular C-terminal end of this receptor, but its precise function in this context is not yet understood [157,159].

WNT ligands, FZD receptors, and the intracellular protein DVL also participate in the β-catenin-independent “non-canonical” WNT pathways [160]. These pathways, however, divert at this level and possess alternative co-receptors, such as the single-pass transmembrane receptor tyrosine kinase-like orphan receptor (ROR1/2) and receptor-like tyrosine kinase (RYK), as well as alternative intracellular effectors including the small GTPases RHO, RAC, and CDC42; c-JUN N-terminal kinase (JNK, *Mapk8*); and the AP-1 complex in the WNT/PCP pathway. In the WNT/Ca^2+^ pathway, the release of intracellular Ca^2+^ causes the activation of Ca^2+^-dependent protein kinases, such as protein kinase C and Ca^2+^/calmodulin-dependent protein kinase II, and of the Ca^2+^-dependent phosphatase Calcineurin, and subsequently of the nuclear factor of activated T cells (NFAT) family of TFs [160,161]. The WNT/PCP and WNT/Ca^2+^ pathways are mostly implicated in the acquisition of cell polarity, morphogenetic (convergent extension) movements, cell migration and neurite extension, and appear to antagonize the WNT/β-catenin pathway in several instances. These “non-canonical” WNT signaling pathways have therefore been proposed to be re-named as “cell polarity” pathways [158]. However, the proliferation or acquisition of a particular fate by, and polarization or migration of, one and the same cell are most likely strongly interconnected, suggesting that a strict separation of both processes and WNT signaling pathways during mammalian development is not possible in many cases.

#### 2.3.2. WNT Signaling in Mammalian mDA Neuron Development In Vivo

The first indication that WNT signals might play a role in the generation of bona fide mDA neurons came from a study showing that both *Wnt1* and *Wnt5a* are expressed in the VM of the midgestational mouse embryo, and that the treatment of primary cells derived from the rodent VM with WNT1- or WNT5A-conditioned media promotes the generation of TH- and NURR1-positive mDA neurons in vitro ([152]; reviewed in Reference [162]). Whereas WNT1-conditioned media appeared to increase primarily the proliferation of mDA progenitors and to a lesser extent their differentiation into mature mDA neurons in these cultures, WNT5A-conditioned media did not affect the proliferation of VM progenitors but enhanced to an even greater extent than WNT1 their differentiation into *Pitx3*- and TH-expressing mDA neurons [152]. Subsequent in vivo analyses of *Wnt1* LOF (*Wnt1* null, *En1-Cre* conditional, and *swaying* hypomorphic) mutant mice revealed an initial (at E11.5) strong reduction and later (after E12.5) complete loss of PITX3- and TH-positive mDA neurons in the *Wnt1* null and conditional mutant VM [153,163], and a preferential loss of VTA DA neurons in the hypomorphic mice ([79]; reviewed in Reference [18]). Moreover, laterally (in the BP) positioned TH-positive but PITX3-negative cells in the *Wnt1* null and conditional mutant VM were reported as ectopically positioned mDA neurons in these mutants [163,164]. Because of the recent finding that several genes used as *bona fide* “mDA markers,” especially *Foxa1*/*2*, *Lmx1a*/*b*, and *Nurr1*, are also expressed in the rostral diencephalon (p3 domain) and particularly in developing hypothalamic neurons [76,77], it might well be that these TH-positive cells are not genuine mDA neurons but rather belong to the diencephalic (hypothalamic) DA groups [165]. In contrast to the *Wnt1* mutant mice, the *Wnt5a* null mutants showed the opposite phenotype: a transient increase of TH-positive mDA neurons at E14.5 that is preceded by an increased proliferation of FP progenitors and increased numbers of NURR1-positive mDA precursors but delayed differentiation of these precursors into TH- and NURR1-expressing mDA neurons at E12.5 ([154]; reviewed in Reference [162]). In addition, a shortening of the A/P and broadening of the mediolateral midbrain axis as well as a disturbed apico-basal orientation of the VM neuroepithelial cells indicated a convergent extension and cell polarity defect in the *Wnt5a* null embryos [154]. Purified WNT5A protein indeed acts in the WNT/PCP pathway via DVL phosphorylation via CK1 and subsequent RAC1 activation in DA cells [154,166,167]. These results suggested that during mDA neuron development in vivo, WNT1 is the more important WNT controlling the generation of mDA progenitors and precursors, and their correct differentiation into mDA neurons. Moreover, they also suggested that WNT1 activates the WNT/β-catenin (“cell fate”) pathway, whereas WNT5A stimulates the WNT/PCP (“cell polarity”) pathway, and that both WNTs partly synergize with and antagonize each other during this process. Ensuing analyses of *Wnt1* and *Wnt5a* double null mutant and conditional β-catenin (because *Ctnnb1* null embryos die very early [168]) mutant mice corroborated these assumptions (reviewed in References [17,162]. The proliferative defect in the *Wnt1* null VM is partly rescued by the simultaneous loss of *Wnt5a* in the *Wnt1*/*Wnt5a* double null embryos, whereas the strong reduction of TH/NURR1-positive mDA neurons in the *Wnt1* null embryos and the midbrain morphogenesis defects of the *Wnt5a* null mutants are both potentiated in the *Wnt1*/*Wnt5a* double null mutants [164]. The conditional β-catenin LOF (using *Shh-Cre*, *Th-IRES-Cre*, and *R26-CreERT* drivers) or GOF (by deleting the N-terminal β-catenin phosphorylation sites using *Shh-Cre*, *Th-IRES-Cre*, and *En1-Cre* drivers) mutant mice, on the other hand, show a decrease or increase and even ectopic generation in the rostral hindbrain, respectively, of NURR1-positive mDA precursors and TH/PITX3-expressing mDA neurons [135,169,170,171,172,173]. Notably, the conditional deletion of β-catenin also affects the β-catenin-containing (bound to cadherins) adherens junctions as well as centrosome formation and mitotic spindle orientation and thus the integrity and polarity of the mutant VM tissues [172,174], revealing the intricate functional relationships between WNT signaling and other intracellular components during development. Similarly, the “artificial” stabilization of β-catenin because of the deletion of the N-terminal phosphorylation sites, leading to a probably excessive WNT/β-catenin signaling in these mutant mice, disrupts the normal differentiation of NURR1-positive mDA precursors into TH- and PITX3-expressing mDA neurons [135,171,173]. The conditional β-catenin mutants thus highlighted the need of a precisely balanced WNT/β-catenin signal for the proper specification of proliferating mDA progenitors and their differentiation into mature mDA neurons.

Consistent with the findings described above, mouse mutants for other components of the WNT/β-catenin signaling pathway expressed in the murine VM, such as the “canonical” WNT2 and WNT7A ligands and the LRP6 co-receptor, revealed a persistent (*Wnt2* null mice) or transient (*Wnt7a* and *Lrp6* null mice, probably due to the compensation by other WNTs/LRPs) reduction of NURR1-positive mDA precursors and TH-positive mDA neurons at late midgestational stages [175,176,177]. Conversely, single and double null mutant mice for the FZD3 receptor, a ubiquitously expressed *Fzd* receptor in neural tissues [178,179], and FZD6 receptor, a *Fzd* receptor that is specifically expressed in the caudal VM of the midgestational mouse embryo [178,179], exhibit mDA and midbrain phenotypes. These alterations are consistent with the known function of these receptors in the WNT/PCP pathway [161,180]. Mutants show a transient reduction of TH-positive mDA neurons at E12.5 that recovers at E13.5 and a mediolateral broadening of the mDA domain is evident in the *Fzd3* null embryos, whereas *Fzd6* null embryos do not show any obvious mDA phenotype, and *Fzd3*/*Fzd6* double null mutants present with a severe midbrain morphogenesis defect and broadening of the mDA domain but otherwise normal numbers of mDA neurons [181]. Accordingly, mouse null mutants for *Sfrp1* or *Sfrp2*, the only two *Sfrp* genes expressed in the murine VM/mDA domain, do not exhibit any defects in mDA neuron development, whereas *Sfrp1*/*Sfrp2* double null mutant mice have an almost identical phenotype to the *Wnt5a* null mutants, suggesting that these WNT/β-catenin antagonists promote WNT/PCP signaling in the mouse VM [182]. Mouse null mutants for the *Ryk* co-receptor, in contrast, show a subtle (20–32%) but persistent reduction of NURR1-positive mDA precursors and TH-positive mDA neurons [183], suggesting that this receptor might also participate in the transduction of a “canonical” WNT/β-catenin signal.

Most of the mouse mutants mentioned above have the caveat of very early and extensive deficits affecting the development of the entire midbrain region and thus potentially masking more specific and/or subtle defects in mDA neuron generation. Therefore, analyses of mouse mutants with no obvious developmental phenotype provided the first indication that a dose-dependent WNT/β-catenin signaling is implicated, in particular, in the subset-specific differentiation of mDA neurons. *Dkk3* is a divergent member of the DKK family of WNT inhibitors expressed in the midline of the VM (medial FP) [184], whose precise function in WNT/β-catenin signaling remains debated [185]. *Dkk3* null mutant mice show a subtle but clear mDA phenotype: a consistent reduction by approx. a fifth (20%) of a TH- and PITX3-positive mDA neuron subset located in the dorsomedial SNc and parabrachial pigmented nucleus (PBP, a part of the VTA) from E12.5 on, and an initially corresponding increase of TH-positive but PITX3-negative cells and later (at E18.5) loss of these and of TH-negative/PITX3-positive cells in this brain region [184]. These deficits are not accompanied by any patterning, proliferation, or specification defects in the *Dkk3* null VM [184], strongly suggesting that the lack of DKK3 and concomitantly of an attenuation of WNT/β-catenin signaling in the medial FP specifically affects the differentiation of a rostrolateral mDA neuronal subset that might be considered the murine equivalent of the human SNc [186]. Generation of the more caudally located VTA DA neurons, probably exposed to higher levels of WNT/β-catenin signaling along the A/P axis of the murine VM [187], is in fact more affected in the conditional β-catenin LOF mutants [135,163,172], whereas the generation of the rostrolateral SNc DA neurons is more affected in the conditional β-catenin GOF mutant mice [135,170,171,173]. Consistent with this idea, the extent and size of the mDA progenitor and mature neuron population in the embryonic mouse VM is also modulated by *miR-135a2*, a microRNA (miRNA) that targets the homeodomain TF *Lmx1b* upstream of the WNT1/β-catenin signaling pathway [188].

Altogether, the in vivo data revealed that “canonical” WNT/β-catenin signaling, prominently activated by the WNT1 molecule that is expressed at the MHB and in the VM, plays a pivotal role during the entire span of mDA neuron development: from the early establishment (patterning) of the mDA progenitor domain in the mammalian VM (not discussed here, see Reference [18]), the intermediate specification of the mDA cell fate in mitotic and postmitotic VM neural precursors, to their later differentiation into mature mDA neurons. Furthermore, they also indicated that a precise and potentially mDA neuron subset-specific balance of the strength of WNT/β-catenin signaling appears to be crucial for the proper generation of mDA neurons in general and of subset-specific (e.g., SNc or VTA) mDA neurons in particular. The “non-canonical” WNT/PCP pathway, in turn, is implicated in the proper morphogenesis of the entire midbrain region, including the establishment of cell polarity and the correct migration and neurite extension within the mDA domain.

#### 2.3.3. WNT Signaling in the Directed Differentiation of PSCs and Direct Conversion of Somatic Cells into mDA Neurons In Vitro

In contrast to the rodent or human embryo in vivo, primary VM or PSC cultures in vitro offer the significant advantage of being easily accessible and manipulable. Initial experiments using rodent primary neuroectodermal tissues (neural plate explant cultures) or VM NSCs showed that *Wnt1* is required to induce ectopic mDA neurons even if FGF8 and SHH, two other essential morphogens for mDA neuron induction [66] (Section 2.1 and Section 2.2), are present, and that WNT1 protein or conditioned media promote the generation of TH-positive mDA neurons [135,152,153]. Conversely, the silencing of *Wnt1* expression in differentiating human multipotent NSCs results in lower yields of TH-positive neurons [189]. Later experiments revealed a ligand- and dose-dependent action of WNT signaling on the differentiation of mDA neurons from rodent and human NSCs or mouse PSCs, whereas treatment of the differentiating cells with WNT5A or other WNT/PCP signaling agonists (such as SFRP1/2) generally increases the yield of TH-positive mDA neurons, addition of WNT1 or of WNT/β-catenin agonists (such as the small molecule GSK3β inhibitors CHIR/CT99021 and kenpaullone, [190]) promotes the generation of TH-positive mDA neurons at low dosage but inhibits it at high dosage, thus confirming the previous in vivo data also in vitro [164,173,191,192,193,194]. Because the derivation of functional and pure (without other contaminating neuronal cell types) mDA neurons turned out to be much more difficult to be achieved from primate and human PSCs, a major breakthrough was the discovery that these PSCs are efficiently differentiated into functional mDA neurons by a protocol that uses the “dual SMAD” inhibitors to suppress alternative mesodermal and endodermal cell fates, and a high dose of SHH or SHH agonists and of the GSK3β inhibitor CHIR99021 in the presence of FGF8 to induce mDA precursors ([43]; reviewed in References [155,156]). However, other groups subsequently noted a concentration- and time-dependent action of WNT/β-catenin pathway activation (i.e., GSK3β inhibition by the CHIR/CT99021 compound) in human and primate PSCs, whereas low CHIR/CT99021 concentrations (i.e., low WNT/β-catenin signaling levels) and/or late application of this compound induces forebrain neurons expressing typical markers for this brain region, high CHIR/CT99021 concentrations (i.e., high WNT/β-catenin signaling levels), and early application of this molecule generates neurons with a hindbrain identity, and only the early application of intermediate CHIR/CT99021 concentrations (i.e., intermediate WNT/β-catenin signaling levels) is capable of inducing genuine mDA neurons that can functionally restore movement deficits in rodent PD models [150,195]. Indeed, recent findings showed that only a precisely titrated and timed addition of the key factors (FGF8, SHH, and a WNT/β-catenin activator/GSK3β inhibitor) for mDA neuron induction from rodent or human NSCs and PSCs during the differentiation procedure is capable of generating fully functional grafts with an authentic mDA neuron identity and without contaminating rostral (forebrain/hypothalamic) or caudal (hindbrain) cells [76,97,196]. Gaucher’s Disease patients carrying mutations in the acid beta-glucocerebrosidase (*GBA1*) gene have a high risk for PD [197], and restitution of the normal WNT/β-catenin signaling levels in human iPSCs derived from these patients (in which this signaling pathway appears to be affected) by CHIR99021 application also improves the generation of mDA neurons from these cells [198]. In agreement with the above-mentioned in vivo data, the appropriate WNT1/β-catenin signaling levels for ensuring the efficient and correct differentiation of mDA neurons from naïve and “primed” (epiblast-derived stem cells) PSCs appear to be regulated by another miRNA, *miR-34b*/*c* [199]. Notably, overexpression of this miRNA also facilitates the direct conversion (so-called “reprogramming” or transdifferentiation) of mouse embryonic fibroblasts (MEFs) into DA-secreting and spontaneously firing induced DA (iDA) neurons, whereas the simultaneous overstimulation of the WNT/β-catenin pathway with the GSK3β inhibitor CHIR99021 suppresses this process [199]. In other contexts, however, application of intermediate CHIR/CT99021 concentrations (i.e., intermediate WNT/β-catenin signaling levels) promotes the direct conversion of MEFs and adult mouse tail tip fibroblasts or human astrocytes into iDA neurons with a typical mDA marker expression and electrophysiological properties using either the four Yamanaka pluripotency TFs *Oct4* (*Pou5f1*), *Sox2*, *Klf4*, and *c-Myc* [200], or the mDA-specific factors *Neurod1*, *Ascl1* (*Mash1*), *Lmx1a*, and *miR-218* [201]. Altogether, these in vitro data indicate that a precise dosage and timing of WNT/β-catenin signaling activation in rodent and primate (including human) PSCs or somatic cells facilitates their differentiation into DA neurons with molecular and physiological characteristics of genuine mDA neurons in vivo and capable of restoring the motor function in animal models of PD.

Most of these protocols did not focus on the generation of specific mDA neuron subsets, although several authors reported that at least part of the generated mDA neurons express molecular markers and/or electrophysiological properties of the rostrolateral SNc DA neurons [43,195,199,200,201]. Using a very similar differentiation protocol as Kriks et al. [43], the combined treatment of differentiating mouse PSCs with WNT1 and DKK3 proteins instead of the potent GSK3β inhibitor CHIR99021 increases the overall proportion of TH-, NURR1-, and PITX3-positive mDA neurons in these cultures [184]. Notably, the differentiating PSCs preferentially adopt a molecular identity of rostrolateral SNc DA neurons expressing KCND3 but not calbindin 1 (CALB1) under these conditions [184]. The expression of the A-type voltage-gated potassium channel subunit KCND3 (Kv4.3) is restricted to SNc DA neurons in the adult rodent VM [202,203], whereas the calcium-binding protein CALB1 (CalbindinD28K) is expressed prominently but not exclusively in VTA DA neurons [8,204]. Despite the still unclear function of DKK3 in the WNT/β-catenin signaling pathway [185], transcriptome profiling of the differentiated PITX3-positive cells strongly suggested that DKK3 attenuates β-catenin-mediated WNT1 signaling to enable the proper generation of rostrolateral (dorsomedial SNc and PBP) mDA neurons, which are selectively affected in the corresponding mouse mutants [184]. Collectively, these data suggest that a fine-tuned activation or attenuation of WNT1/β-catenin signaling might even enforce the generation of a particular mDA neuron subset, VTA or SNc, from murine and human PSCs or somatic cells.

### 2.4. BMP Signaling in Midbrain Dopaminergic Neuron Generation In Vivo and In Vitro

#### 2.4.1. The BMP Signaling Pathway

BMPs are part of the TGFβ protein superfamily. The TGFβ family members are subdivided into two groups: the BMP group that includes all BMPs and most growth and differentiation factors (GDFs) and the TGFβ group that comprises the three mammalian TGFβ isoforms (TGFβ1–3), activins, nodals, and some GDFs.

BMPs were first identified as proteins that induce ectopic bone formation, hence their name [205]. BMPs regulate a wide array of neurodevelopmental processes, including progenitor proliferation, apoptosis, and differentiation [206,207,208]. Depending on the cell type, extracellular environment and developmental stage, they might enhance or inhibit these processes. Over a dozen BMPs have been identified in vertebrates so far [205]. BMP family members can be classified into several subgroups according to their structural similarities. These subgroups include the BMP2/4 group, BMP5/6/7 (OP1)/8 group, BMP9/10 group, and BMP12/13/14 group [205]. Specifically, the two subgroups which were named after their Drosophila orthologs, the decapentaplegic (DPP) subgroup (BMP2 and BMP4), and the 60A subgroup (BMP5, BMP6, BMP7, and BMP8), are expressed in the mammalian nervous system [209,210], with the exception of BMP8, which is not expressed in the brain [211].

BMPs are secreted proteins that bind to BMP receptors (BMPRs) as homodimers or heterodimers. The heteromeric BMPR complexes contain type I and type II serine-threonine kinase receptor subunits (Figure 2). BMPs are capable of binding to type I receptors in the absence of type II receptors. However, the presence of type I and type II receptors significantly increase the binding affinities of BMPs [205,212]. The type II receptors phosphorylate and thereby activate the type I receptors, which transduce the BMP signal. In mammals, three type II receptors mediate the activity of BMPs: the BMP type II receptor (BMPRII), the activin type II receptor (ACTRII), and the activin type IIB receptor (ACTRIIB). In contrast to BMPRII, which is specific for BMPs, ACTRII and ACTRIIB are activated by BMPs, activins, and myostatin [205,212]. The activin receptor-like kinases (ALK) 1, 2, 3 (BMPRIA), and 6 (BMPRIB) serve as type I receptors for most BMPs. In addition to ligand-receptor specificity, BMP signaling can be further regulated extracellularly by direct interaction of BMPs with their secreted antagonists such as noggin (NOG) (Figure 2) [205].

The canonical BMP signaling pathway is activated by binding of BMPs to BMPRs and mediated by SMADs (Figure 2). Activated type I receptors signal by phosphorylating the receptor-regulated SMADs (R-SMADs), SMAD1, SMAD5, and SMAD8, which can then bind the co-regulated SMAD4 (coSMAD). The R-SMAD/coSMAD complexes translocate and accumulate in the nucleus, where they act as TFs and participate in the regulation of target gene expression [213] (Figure 2). Phosphorylated SMAD1, 5, and 8 are the major intracellular BMP signaling pathway components. BMPs also activate SMAD-independent signaling pathways such as MAPKs, JNK, PI3K, AKT, and small GTPases [205,214]. These non-canonical pathways cooperate with SMAD pathways to regulate various cellular responses. In contrast to the BMP group members, TGFβ group members bind to TGFβ and activin type II receptors and use predominantly ALK4, 5, and 7 as type I receptors, which signal via SMAD2 and SMAD3 [213].

#### 2.4.2. The Function of the BMP Signaling Pathway in the Generation of Mammalian mDA Neurons In Vivo

In contrast to WNTs, FGFs, and SHH, the essential role of BMPs in the formation of mammalian mDA neurons in vivo has only been discovered recently [19]. The first indication that BMP signaling could be involved in the generation of mammalian mDA neurons in vivo came from in vitro studies [215,216] and expression studies in mice [19]. Before the onset of mDA neurogenesis at E10.5, BMP5, BMP6, and BMP7, as well as phosphorylated SMAD1/5/8 (pSMAD1/5/8), are expressed in the VM close to and within the mDA progenitor domain. The BMP receptor 1B (BMPR1B), which plays a critical role in neuronal differentiation [217], is confined to the mDA domain around the peak of mDA neurogenesis at E12.5 [19].

To assess the function of BMP signaling in the development of mDA neurons, the formation of these cells were analyzed in different *Bmp* single and compound LOF mouse mutants [19]. Changes were not apparent in the generation of mDA neurons in *Bmp5*, *Bmp6*, and *Bmp7* single-null mutants, nor in *Bmp5*/*Bmp6* and *Bmp6*/*Bmp7* double-null mutants. In *Bmp5*/*Bmp7* double-null mutants, however, postmitotic mDA neurons, expressing NURR1 and the pan-neuronal marker βIII tubulin (TUJ1, *Tubb3*), were entirely absent at E10.5, the latest developmental time point mutants can be studied before they die of cardiac malformations. Different mechanisms are used by BMP5/7 to control the generation of mDA neurons. First, BMP5/7 regulate the proliferation of the mDA progenitor population. Second, BMP5/7 promote mDA neurogenesis by regulating MSX1/2 and NGN2 expression. In contrast to the mDA progenitor domain within the FP, BMP5/7 appear to prevent the premature neurogenesis in the adjacent midbrain BP. As discussed below, these differences can be explained by the expression of SHH in the FP, which is under the regulatory control of BMP5/7.

Based on the lack of postmitotic mDA neurons and the downregulation of pSMAD1/5/8 in *Bmp5*/*Bmp7* double null mutants, it was hypothesized that components of the SMAD signaling pathway are mediating aspects of the effect of BMP5/7 on mDA neuron generation [19]. To test this hypothesis, the formation of mDA neurons was assessed in mutants in which *Smad1* was conditionally inactivated starting from approximately E10−11 using a *Nestin-Cre* driver [218]. Consistent with a role in mediating the effects of BMPs on mDA generation, the conditional inactivation of *Smad1* in mouse NSCs in vivo leads to a significant reduction of mDA neurons. Although *Bmp5*/*Bmp7* double null mutants show a decrease in mDA progenitor cell proliferation at E10.5, conditional *Smad1* mutants show an increase at E12.5. The downregulation of pSMAD1/5/8 and the unaltered expression of the non-canonical BMP pathway in *Bmp5*/*Bmp7* double null mutants suggest that the differences in cell proliferation are unlikely to be caused by disruptions of different signaling pathways. Instead, the different phenotypes might be explained by the different time points when the consequences of *Bmp*/*Smad* inactivation were studied. This suggests a distinct response of forming mDA neurons to the BMP/SMAD pathway at different stages of their development.

Evidence that the BMP receptors BMPR1A and BMPR1B have sequential roles during development might explain the phenotypic differences between *Bmp5*/*Bmp7* double null and conditional *Smad1* mutants [217]. BMPR1A is ubiquitously expressed in NSCs from early development onward and regulates the proliferation of these cells. In contrast, BMPR1B starts to be expressed only later during embryogenesis, causing mitotic arrest and terminal differentiation [217]. At the onset of mDA neurogenesis at E10.5, the reduced BMPR1A/SMAD activation in *Bmp5*/*Bmp7* double null mutants is responsible for the reduced cell proliferation. During later stages (at E12.5), when SMAD1 mediates the differentiating effects of BMPR1B, whose expression is confined to the mDA progenitor domain in the VM [19], loss of this signaling component attenuates the cell cycle exit and differentiation of mDA neurons.

Interestingly, TH-/SOX6-positive and TH-/GIRK2-positive SNc DA neurons were especially reduced in the conditional *Smad1* mutants [19]. In contrast, the red nucleus neurons developed normally in the BP of these mutants. Further experiments studying the molecular underpinnings of this specific vulnerability of SNc DA neurons to reduced or absent BMP signaling are expected to provide important information for the better understanding of the specific degeneration of SNc DA neurons in PD.

#### 2.4.3. BMPs in Stem Cell-Derived mDA Neuron Maturation

Current protocols for the derivation of mDA neurons from mammalian PSCs are based on the activation of the FGF, SHH, and WNT signaling pathways, which regulate the generation of mammalian mDA neurons in vivo [12,42,43,44]. In contrast, the potential of BMPs in the differentiation of PSCs to mDA neurons has not been investigated extensively and the few publications did not yield consistent conclusions so far. Thus, early exposure to BMP4 in a protocol for mDA neuron differentiation from human ESCs results in a significant reduction of DA neurons [219]. In contrast, early addition of BMP2, together with SHH and FGF8, promotes the differentiation of mDA neurons by modulating the SHH gradient and increasing the expression of LMX1A and FOXA2 in human PSCs [220]. More recently, BMP7, together with pramipexole, have been reported to increase the yield of DA neurons differentiated from forebrain-derived human NSCs [221].

A significant progress in the directed neural differentiation of human PSCs was the discovery that blocking the BMP/TGFβ (”dual SMAD”) signaling pathway during the initial steps of the protocol leads to a highly efficient neural conversion [42,43,44]. However, the role of BMPs during the later stages of mDA specification and maturation in vitro remained unclear. To address this issue, the potential of BMP5/7 was studied in the directed differentiation of mDA neurons from two independent human iPSC lines and a previously established induced NSC (iNSC) line [222,223,224,225]. For this purpose, a widely used mDA differentiation protocol was modified that includes the activation of the FGF8 and SHH signaling pathways [226]. BMP5/7 treatment during the 22 days of the maturation phase resulted in a strong increase in the numbers of TH-positive neurons derived from both human iPSC lines and iNSC cultures [19]. Conversely, the requirement of BMP5/7 for the generation of mDA neurons was tested by blocking the BMP signaling pathway using NOG in two independent human iPSC lines during the maturation phase. The number of TH- and TUJ1-positive mDA neurons is significantly reduced after NOG treatment in both cultures, indicating a blockade of mDA neuron generation [19]. Taken together, these data indicate that BMP5 together with BMP7 robustly promote the maturation of PSCs and NSCs into mDA neurons, and that activation of the BMP signaling pathway during the maturation phase is an essential part of currently used protocols for the generation of mDA neurons from mammalian PSCs.

### 2.5. Cross-Talk between the FGF, SHH, WNT, and BMP/TGFβ Signaling Pathways in Midbrain Dopaminergic Neuron Generation In Vivo and In Vitro

#### 2.5.1. Crosstalk between FGF/FGF8 and WNT Pathways

FGF and WNT signaling pathways interact during early midbrain development to establish and maintain the activity of the IsO. Experiments both in vivo and in vitro have shown that the two signaling molecules regulate the expression of each other, resulting in the complementary patterns of *Fgf8* and *Wnt1* expression on the opposite sides of the MHB [227,228,229,230,231]. The expression of *Fgf8* is lost when WNT1 signaling is inactivated and, conversely, ectopic WNT stimulation or activation of the β-catenin mediated “canonical” WNT signaling pathway can increase *Fgf8* expression. Interestingly, stabilization of β-catenin results in *Fgf8* upregulation in the anterior hindbrain (rhombomere 1), but not in the midbrain, demonstrating different competence of these brain regions and providing a partial explanation for the complementary expression patterns [169,230,231]. On the other hand, FGF signaling is also thought to maintain, rather than induce, *Wnt1* expression in the posterior midbrain [232]. Although not completely understood, these cross-regulatory mechanisms may include direct or indirect transcriptional control by the signal-dependent TFs. The latter may involve downstream TFs like EN1 [233,234,235,236].

In contrast to the IsO, where the expression of *Wnt1* and *Fgf8* genes depend on each other, *Wnt1* expression is independent of FGF signaling in the mouse VM, which gives rise to the mDA neurons [57]. The *Wnt1* gene may thus have distinct regulatory elements driving expression in the IsO and the VM tissue. This also suggests that the effects of FGF signaling on the mDA progenitors are not due to changes in local *Wnt1* transcription. However, there are other, yet incompletely understood, possibilities for interaction of the FGF and WNT signaling pathways. For example, FGF signaling was recently shown to regulate the translational efficiency of several WNT signaling pathway components in mammary epithelial cells [237]. In addition, regulation of SPRY4, a negative feedback regulator of the RAS-MAPK (mitogen-activated protein kinases) pathway, was suggested to be regulated by both FGF and WNT signaling in the zebrafish dorsal midbrain [231]. Similar cross-regulation of feedback antagonists of WNT and FGF signaling pathways has been observed in the zebrafish lateral line [238]. Studies in other developmental contexts have demonstrated multiple interactions between the signaling cascades downstream of the FGF and WNT receptors, with effects on β-catenin activity, Ca^2+^ signaling, and the cytoskeleton [239]. How such mechanisms operate in the developing mDA neuron progenitors remains unknown.

#### 2.5.2. Crosstalk between SHH and WNT Pathways

As described above, the combined activation of WNT and SHH signaling is crucial for the induction of authentic mDA neurons from human PSCs. Studies on the spinal cord have uncovered some of the molecular mechanisms that could account for the intersection between WNT and SHH signaling [240,241]. In mDA progenitor induction and neurogenesis, LOF and GOF studies in mice provide evidence for the interaction between these two signaling pathways.

Conditional inactivation of *Smo* using *Shh-Cre* results in a subtle increase in *Wnt1* expression, while constitutive activation of SHH signaling in *Shh*-expressing cells leads to a slight reduction in *Wnt1* expression [124] (Figure 2). In contrast, the loss of primary cilia in *Ift88* cKO mutants and the concomitant inactivation of SHH signaling results in reduced expression of *Wnt1* and *Wnt5a* and a reduced expression of the WNT signaling readout *Axin2* [142]. An explanation for the discrepancy in the phenotype between mice lacking primary cilia and mice with inactivated *Smo* might be the time point of inactivation, the spatial extent of inactivation (the entire midbrain or just the FP, respectively), or a direct effect of primary cilia loss on *Wnt* expression and signaling [242]. Thus, while these results suggest that SHH signaling regulates *Wnt* expression in the VM, the precise effect of SHH on Wnt expression levels remains to be explored.

On the other hand, WNT signaling regulates *Shh* expression in the mDA progenitor domain. The downregulation of *Shh* in the ventral midline that is normally observed in this domain at E11.5 does not occur when WNT signaling is abolished in the FP by inactivating β-catenin (*Ctnnb1*) in *Shh*-expressing progenitors. In the converse experiment, the expression of a stabilized β-catenin in the *Shh*-expressing population, *Shh* expression is downregulated in the midbrain FP [135,171] (Figure 2). Moreover, high doses of SHH and WNT antagonize each other’s ability to induce mDA cell fates, both in cultures of VM progenitors and during the induction of mDA neurons from ESCs [173]. Based on these results, it has been proposed that WNT signaling promotes neurogenesis through the downregulation of *Shh* in the mDA progenitor domain [135,173]. Still, SHH signaling itself plays only a minor, transient role in regulating the proliferation and neurogenesis of the mDA progenitors [122,124]. A more recent study dissected the effect of WNT upregulation on mDA neuron development in further detail, demonstrating that activated WNT signaling does not only downregulate expression of *Shh*, but also of *Foxa2* and *Lmx1b*. Ventral midline progenitors adopt a mixed identity, expressing factors characteristic for mDA progenitors (LMX1A/B) along with factors characteristic for red nucleus progenitors (NKX6-1 and NEUROG1). These NEUROG1-expressing progenitors give rise to neurons with features typical of the red nucleus, at the expense of mDA neurons [171]. Thus, while it is clear that WNT regulates *Shh* expression (directly or indirectly), the functional consequence on mDA neuron development in vivo is likely mediated by a more general role of WNT signaling in determining mDA progenitor fate.

#### 2.5.3. Crosstalk between WNT and BMP/TGFβ Pathways

Another potential but only rudimentarily explored crosstalk in mDA neuron development is the interaction between the WNT and BMP/TGFβ signaling pathways. First hints towards such a crosstalk during the in vitro generation of mDA neurons from human PSCs came from a systematic study about the effects of the “dual SMAD” (BMP and TGFβ) inhibitors, now widely used in the human PSC differentiation protocols for mDA neurons [243]. The authors of this study concluded that inhibitors of the BMP/SMAD1, 5, 8 signaling pathway in particular, or the combined treatment with BMP and TGFβ/SMAD2, 3 inhibitors, increase indirectly the expression of *Wnt1* and WNT/β-catenin signaling through the induction of the Zinc finger E-box binding homeobox 2 (ZEB2 and SIP1) TF, which in turn represses the gene encoding the WNT inhibitor *Sfrp1* [243]. However, they also noted that the efficient generation of authentic mDA neurons requires the presence of potent SHH agonists (probably because of the strong repression of *Shh* by the increased WNT/β-catenin signaling, see Section 2.5.2) and of FGF8 in these cultures, and that the administration of the potent GSK3β inhibitor CHIR99021 might not be necessary if the combined BMP/TGFβ inhibitor levels are sufficiently high to induce a strong enough WNT/β-catenin signal in this protocol [243]. Altogether, their data suggested that WNT/β-catenin signaling is antagonized by the BMP/TGFβ pathway during mDA neuron generation in vitro. This finding is in line with previous observations in the dorsal midbrain of conditional TGFβ type II receptor (*Tgfbr2*) mouse mutants [244]. Based on the unaltered expression and distribution of *Wnt1* mRNA and β-catenin protein in the VM of *Bmp5*/*Bmp7* double null and conditional *Smad1* mutants, no obvious evidence for a crosstalk between the BMP/SMAD1 and WNT1/β-catenin signaling pathways was found during mouse mDA neuron development in vivo [19]. However, the significantly reduced levels of phosphorylated (destabilized) β-catenin in the VM of the *Bmp5*/*Bmp7* double null mutants might hint at a corresponding increase of unphosphorylated (stabilized) β-catenin levels and thus activation of the WNT/β-catenin signaling pathway in the absence of BMP5/7 [19]. Because this interpretation would be at odds with the observed increase of SHH expression and reduced proliferation and neurogenesis of mDA progenitors in the *Bmp5*/*Bmp7* double-null mutant VM [19], further experiments are warranted to clarify this issue.

Perhaps the most intriguing clue so far for a potential crosstalk between the WNT/β-catenin and BMP/TGFβ signaling pathways in mDA neuron development is provided by the previous finding that DKK3, a putative inhibitor or at least attenuator of WNT/β-catenin signaling in different tissue contexts [245], not only promotes the proper differentiation of a rostrolateral mDA neuron subset, but also their later survival in the mouse VM and in the culture dish [184,246]. During development (both in vivo and in vitro) and in a genetic mouse model of PD (the heterozygous *En1* mouse, [247]), this is at least partly due to the downstream induction or maintenance, respectively, of a WNT1/β-catenin-driven neuroprotective gene cascade comprising the TFs *Lef1*, *Lmx1a*, and *Pitx3*, and the neurotrophic factors *Bdnf*, *Fgf20*, and *Dkk3* [184,246,248,249,250] (Figure 2). More recent and yet unpublished findings, however, indicate that only very few WNT/β-catenin-responsive mDA neurons are detected within the rostrolateral SNc region at late developmental and adult stages in the mouse, as opposed to the caudomedial VTA DA region, where numerous WNT/β-catenin-responsive mDA neurons reside at these stages [251]. This raises the possibility that DKK3 acts via a completely distinct pathway in this context, namely TGFβ/SMAD signaling, which has been widely implicated in the survival and neuroprotection of developing and mature mDA neurons in the mammalian brain (reviewed in Reference [252]). Because DKK3 can both synergize with and antagonize the TGFβ/SMAD signaling pathway in different cell and tissue contexts [253,254,255,256], the precise mechanism of DKK3 action during mDA neuron development and adult survival remains elusive. Based on the current evidence, however, DKK3 might activate or maintain TGFβ/SMAD signaling in medial FP mDA progenitors and maturing rostrolateral (dorsomedial SNc and PBP) mDA neurons. Moreover, because the LOF of SMAD1 in conditional *Smad1* mutants also affects preferentially the differentiation of rostrolateral (SNc) SOX6- and GIRK2-positive mDA neurons [19], DKK3 might even synergize with SMAD1-mediated BMP signaling in this context. A better understanding of the generation of this particular mDA neuron subset during embryonic development, especially in view of more efficient and consistent PSC differentiation protocols for this mDA neuron subtype, still awaits clarification of these points.

#### 2.5.4. Crosstalk between BMP and SHH Pathways

Since BMPs were only recently identified to play an essential role in the generation of mammalian mDA neurons in vivo, only little is known about the interaction of BMP signaling with other pathways. During the studies of the requirement of BMP5/7 and SMAD1 for the development of mDA neurons, the interactions of the BMP signaling pathway with the WNT and SHH signaling pathways were investigated. To do this, the expression of WNT and SHH signaling components were analyzed in *Bmp5*/*Bmp7* double null mutants.

Neither *Wnt1* mRNA nor total β-catenin protein expression, which plays a critical role in mediating “canonical” WNT/β-catenin signaling, are altered in *Bmp5*/*Bmp7* double null mutants [19]. Moreover, cell adhesion, which is dependent on intact β-catenin in the mDA progenitor domain [172,174], is unperturbed in the double null mutants as assessed by the normal expression of N-cadherin and ZO1 [19]. In contrast, the expression of SHH, measured as a ratio between the SHH immunoreactivity in the midline and the SHH signal in a region outside of the SHH expression domain, is nearly doubled in the *Bmp5*/*Bmp7* double null mutants [19].

There are many examples for a close interaction between the SHH, WNT, and BMP signaling pathways in the generation of non-mDA neuronal populations [257]. Thus, an interaction of all three signaling pathways might provide the permissive environment necessary for mDA neurogenesis. Starting at around E8.0, SHH signaling is important for the formation of the SHH-/FOXA2-positive FP and mDA progenitor domain [66,120,125]. However, at the onset of mDA neurogenesis, SHH was suggested to inhibit FP mDA neurogenesis [135]. The WNT/β-catenin pathway is necessary and sufficient for the restriction of SHH expression. Based on this observation it was suggested that this WNT/SHH interaction creates a permissive environment for mDA neurogenesis [135,172,173]. The upregulation of SHH expression and the concomitant reduced mDA progenitor proliferation and neurogenesis in the *Bmp5*/*Bmp7* double-null mutants therefore indicates that the restriction of SHH availability by BMPs might provide one mechanism by which BMPs control mDA neurogenesis. The property of BMP7 to reduce SHH expression is not limited to the midbrain, as demonstrated by ectopic expression of BMP7 in the hindbrain. Misexpression of BMP7 in this brain region, where it is normally not expressed, attenuates the expression of SHH in the FP [258]. Taken together, these data suggest that one of the functions of the BMP5/7 signaling pathway in the development of mDA neurons is to create a permissive environment for mDA neurogenesis by restricting the expression of SHH in the midbrain FP.

## 3. Conclusions

In this review, we have summarized the current knowledge on the role of the four major signaling pathways and their known or putative interactions regulating the generation of mDA neurons in vivo and in vitro (Table 1). Despite substantial advances in recent years, we are only at the beginning of understanding how the crosstalk between these four prominent signaling pathways orchestrates the development of mDA neurons in the mammalian embryo, and how we can use this knowledge to generate stem cell-derived and subtype-specific mDA neurons.

Many open questions still remain regarding the in vivo and in vitro generation of mammalian mDA neurons and will continue to make it an exciting research field, but the two most pressing are: (1) Which molecular mechanisms underlie the specification of SNc DA neurons versus VTA DA neurons in vivo? This question is of particular interest, as it can be expected that the answer will provide direct insights into the specific vulnerability of SNc DA neurons in PD. (2) What will be the results of the very recently initiated clinical trials using human stem cell-derived mDA neurons for cell replacement therapy? Efficacy and safety outcomes of these trials will have a major impact for the direction of future research. Tailoring essential parameters of cell grafts including the degree of differentiation, cellular composition, and survival rates will require also in the future a close interaction between basic and clinical research.

## Figures and Tables

**Table 1:** Functions and crosstalk of the four main signaling pathways in mDA neuron generation.

	FGFs	SHH	WNTs	BMPs
**VM patterning**	Yes	Yes	Yes	No ^1^
**Progenitor proliferation**	Yes	Yes	Yes	Yes
**Subtype specification**	Yes? ^2^	Yes	Yes? ^2^	Yes
**Differentiation**	Yes	No	Yes	Yes
**Survival**	Yes	No	Yes	N.D. ^3^
**In vitro stem/somatic cell differentiation**	Mouse and human ESCs, iPSCs, iDA	Mouse and human ESCs, iPSCs, iNSC	Mouse and human ESCs, iPSCs, iDA	Human iPSCs, Human iNSC
**Known pathway interaction**	Promotes *Wnt1* expression	Regulates *Wnt* expression	Represses *Shh,* promotes *Fgf8* expression	Represses SHH expression

^1^ VM patterning has only been assessed for BMP5/6/7. ^2^ Final evidence is still missing. ^3^ Not determined.
